# Draft genome sequence and probiotic functional property analysis of *Lactobacillus gasseri* LM1065 for food industry applications

**DOI:** 10.1038/s41598-023-39454-2

**Published:** 2023-07-27

**Authors:** Won-Young Bae, Young Jin Lee, Woo-Hyun Jung, So Lim Shin, Tae-Rahk Kim, Minn Sohn

**Affiliations:** Microbiome R&D Center, Lactomason, Seoul, 06620 Republic of Korea

**Keywords:** Microbiology, Pathogenesis

## Abstract

Probiotics are defined as live organisms in the host that contribute to health benefits. *Lactobacillus gasseri* LM1065, isolated from human breast milk, was investigated for its probiotic properties based on its genome. Draft genome map and de novo assembly were performed using the PacBio RS II system and hierarchical genome assembly process (HGAP). Probiotic properties were determined by the resistance to gastric conditions, adherence ability, enzyme production, safety assessment and mobile genetic elements. The fungistatic effect and inhibition of hyphae transition were studied using the cell-free supernatant (CFS). *L. gasseri* LM1065 showed high gastric pepsin tolerance and mild tolerance to bile salts. Auto-aggregation and hydrophobicity were measured to be 61.21% and 61.55%, respectively. The adherence to the human intestinal epithelial cells was measured to be 2.02%. Antibiotic-resistance genes and putative virulence genes were not predicted in the genomic analysis, and antibiotic susceptibility was satisfied by the criteria of the European Food Safety Authority. CFS showed a fungistatic effect and suppressed the tricarboxylic acid cycle in *Candida albicans* (29.02%). CFS also inhibited the transition to true hyphae and damaged the blastoconidia. This study demonstrates the essential properties of this novel probiotic, *L. gasseri* LM1065, and potential to inhibit vaginal *C. albicans* infection.

## Introduction

*Lactobacillus gasseri*, previously classified as the *Lactobacillus acidophilus* complex^1,2^, is present in the gastrointestinal tract (GIT), oral cavity, vaginal tract^2,3^, and human milk^1,4^. *L. gasseri* is an obligate homofermentative and thermophilic lactic acid bacteria (LAB) strain^2^, with a reported genomic size of approximately 1.89 Mb. *L. gasseri* was initially referred to *as L. acidophilus* and was reclassified as a separate species along with *Lactobacillus johnsonii*^3^. Many researchers have investigated *L. gasseri* as a probiotic, such as a yogurt starter^4^, for the treatment of vaginal dysbiosis^5^, inflammation^6^, oral diseases^7^, and type 2 diabetes^8^. In clinical trials, *L. gasseri* CP2305 has been applied as a heat-inactivated probiotic to study premenstrual symptoms in young women^9^ and to relieve fatigue- and stress-related symptoms in male runners^10^.

Vulvovaginal candidiasis (VVC) is a type of genital infection in women caused by excessive *Candida* genus abundance and imbalance of vaginal microbiome in the reproductive phase^11–14^. VVC leads to vulval discomfort and pain accompanied by pruritus, vaginal soreness, and abnormal vaginal discharge^12^. According to a previous study, VVC can frequently occur in adult women in their lifetime, and 80–90% of VVC is caused by *Candida albicans*^11,14^. Despite the high frequency of VVC pathogenesis and recurrent vulvovaginal candidiasis (VVCR), *Candida* infections in the vaginal tract have not been clearly elucidated. *Candida* species, including *C. albicans*, is considered to migrate (like vaginal *Lactobacillus* species) from the lower GIT to the vaginal tract^13^. Amphotericin B and nystatin family of polyene antifungal agents are commonly used to treat VVC^11,14^. However, these antifungal agents affect the microbial environment in the vaginal flora^12^ and may also lead to the development of VVCR in VVC patients^11^.

In this study, we aimed to isolate a novel probiotic strain and investigate its probiotic properties based on genomic information. Many probiotic strains have been investigated for their probiotic properties, such as resistance to gastric and intestinal conditions, enzyme production, adhesion to intestinal cells, and safety. Nevertheless, these properties are needed at the genomic level for probiotics. In this study, *L. gasseri* LM1065 isolated from human breast milk was investigated for its essential probiotic properties and antifungal abilities against *C. albicans*, along with functional gene annotation.

## Results

### General genomic feature of *Lactobacillus gasseri* LM1065

**Figure 1:**
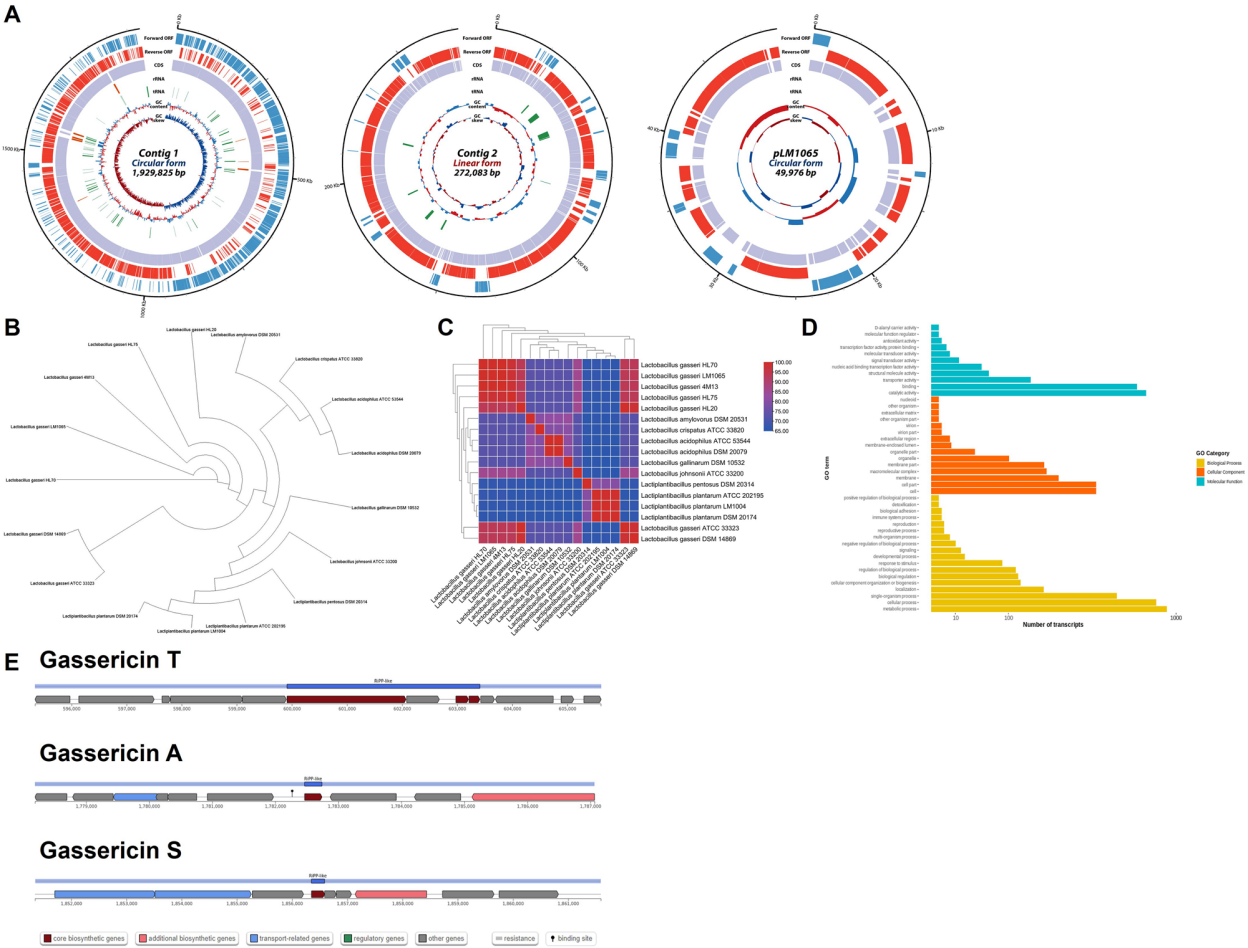
Circular gene map, phylogenetic relationship, and Gene Ontology of *Lactobacillus gasseri* LM1065. (**A**) Circular gene map of three contigs. Each circle from outside to inside indicates protein-coding sequences (CDS) on forward strand, CDS on reverse strand, tRNA, rRNA, GC content, and GC skew. (**B**) Phylogenetic tree. (**C**) Average nucleotide identity (ANI) value. (**D**) Gene Ontology. (**E**) Bacteriocin gene cluster.

As shown in Fig. 1A, the genomic analysis of *L. gasseri* LM1065 comprises two contigs (circular form and linear form, respectively) and a circular plasmid. The size of the entire genome sequence of *L. gasseri* LM1065 was 2,251,884 bp. The plasmid of *L. gasseri* LM1065 was similar to plasmid of *L. gsseri* HL70. The whole genome of *L. gasseri* LM1065 showed a 35.02% GC content. The GC contents of each contig were 34.98% (circular contig), 35.09% (linear contig), and 35.99% (plasmid). In total, 2300 protein-coding sequences (CDS) were identified in *L. gasseri* LM1065. Contig 1 (circular contig) was identified in 1920 CDS, and contig 2 (linear contig) and plasmid contained 322 and 58 coding sequences, respectively. The *L. gasseri* LM1065 contained 15 rRNA and 79 tRNA genes. Most rRNA and tRNA genes were identified in contig 1, and one tRNA was found in contig 2. This Whole Genome project has been deposited at DDBJ/ENA/GenBank under the accession JAQOUF000000000.

### Phylogenetic relationship of *Lactobacillus gasseri* LM1065

The ortholog phylogenetic relationships of *L. gasseri* LM1065 among *the Lactobacillus* and *Lactiplantibacillus* are shown in Fig. 1B and C. According to the phylogenetic network based on ortholog (Fig. 1B), *L. gasseri* LM1065 was grouped into other *L. gasseri* and further associated with *L. acidophilus* complex. In the average nucleotide identity (ANI) level comparison, *L. gasseri* LM1065 was relatively close to other *L. gasseri* (93.51–99.93). The *L. gasseri* strains including *L. gasseri* LM1065 showed approximately 85% of ANI distance with *L. johnsonii* ATCC33200 and other *L. acidophilus* complex strains showed approximately 73% of distance. *Lactiplantibacillus*, which are different species, showed approximately 65% of ANI distance (Fig. 1C).

### Genomic insight into *Lactobacillus gasseri* LM1065.

Figure 1D shows the predicted gene cluster into different functional categories (biological processes, cellular components, and molecular functions) depending on the Gene Ontology (GO) database. Total 6624 of transcripts were annotated and divided into 18 biological processes, 15 cellular components, and 11 molecular function categories. In total, 2300 CDS in all contigs, adhesion ability, bacteriocin production, enzyme production, and stress response functional genes are shown in Table 1.

**Table 1:** Probiotic property-related functional gene annotation of *Lactobacillus gasseri* LM1065.

Genome assembly statistics	Value		
Total length (bp)	2,251,884		
Largest contig length (bp)	1,929,825		
N50 length (bp)	1,929,825		
Number of contig	3		
GC contents (%)	35.02		
Protein-coding sequences	2300		
Total rRNA	15		
Total tRNA	80		

Function	Gene product	E-value	GO name (ID)
Adhesion in gastrointestinal tract	LPXTG cell wall anchor domain-containing protein	0	
	Adhesin	1.8E−272	
	D-alanyl-lipoteichoic acid biosynthesis protein	1.2E−251	P:GO:0046486; C:GO:0005737; F:GO:0046872; P:GO:0019350; F:GO:0047348
	D-alanine—poly(phosphoribitol) ligase	2.1E−289	
	Fibronectin-/fibrinogen-binding protein	0	F:GO:0047473; F:GO:0005524; F:GO:0016208; C:GO:0005737; P:GO:0070395; P:GO:0046436
Auto-aggregation	Exopolysaccharide biosynthesis protein	1.6E−148	P:GO:0006570; F:GO:0004725; F:GO:0030145; P:GO:0035335
Bacteriocin production	CPBP family intramembrane metalloprotease	9.4E−219	
	Gassericin T subunit (lactacin F family)	1.2E−19	
	Gassericin T subunit (GatX)	4.5E−30	P:GO:0042742
Enzyme production	1-Acyl-sn-glycerol-3-phosphate acyltransferase	3E−163	F:GO:0016746; P:GO:0008152; F:GO:0016798
	Aminopeptidase	8.7E−255	P:GO:0006508; F:GO:0004197; F:GO:0004177
	β-Galactosidase	0	P:GO:0046486; F:GO:0046872; C:GO:0009341; P:GO:0006012; F:GO:0004565; P:GO:0006027; P:GO:0006687
	β-Glucanase	3.2E−213	P:GO:0005975; F:GO:0004553
	Glycoside hydrolase family 1	3.6E−305	P:GO:0046486; C:GO:0009341; P:GO:0006012; F:GO:0004565; P:GO:0006027; P:GO:0006687
	Prolyl aminopeptidase	1.6E−178	P:GO:0006508; F:GO:0004177
Stress response	Acid shock protein	5.2E−33	
	Cardiolipin synthase	3.7E−272	F:GO:0008808; C:GO:0016021; C:GO:0005886; P:GO:0032049; F:GO:0016787
	Flavocytochrome c	0	
	Hsp20 alpha crystallin family protein	1.2E−72	P:GO:0006950
	Hsp33 family molecular chaperone	7.5E−162	C:GO:0005737; F:GO:0051082; P:GO:0006457; P:GO:0006950
	Sodium-proton antiporter	1.7E−273	F:GO:0015299; P:GO:1902600; C:GO:0016021; P:GO:0006885
	Universal stress protein	1E−86	C:GO:0005737; P:GO:0006950

### Prediction of gassericin gene cluster

Figure 1E shows bacteriocin production gene clusters of *L. gasseri* LM1065. Total three bacteriocin gene clusters were predicted in contig 1. Gassericin T (start at 595,336 and end at 605,613), gassericin A (start at 1,778,187 and end at 1,787,064) and gassericin S (start at 1,851,350 and end at 1,861,589) were predicted in *L. gasseri* LM1065.

### Comparative genomic analysis

**Figure 2:**
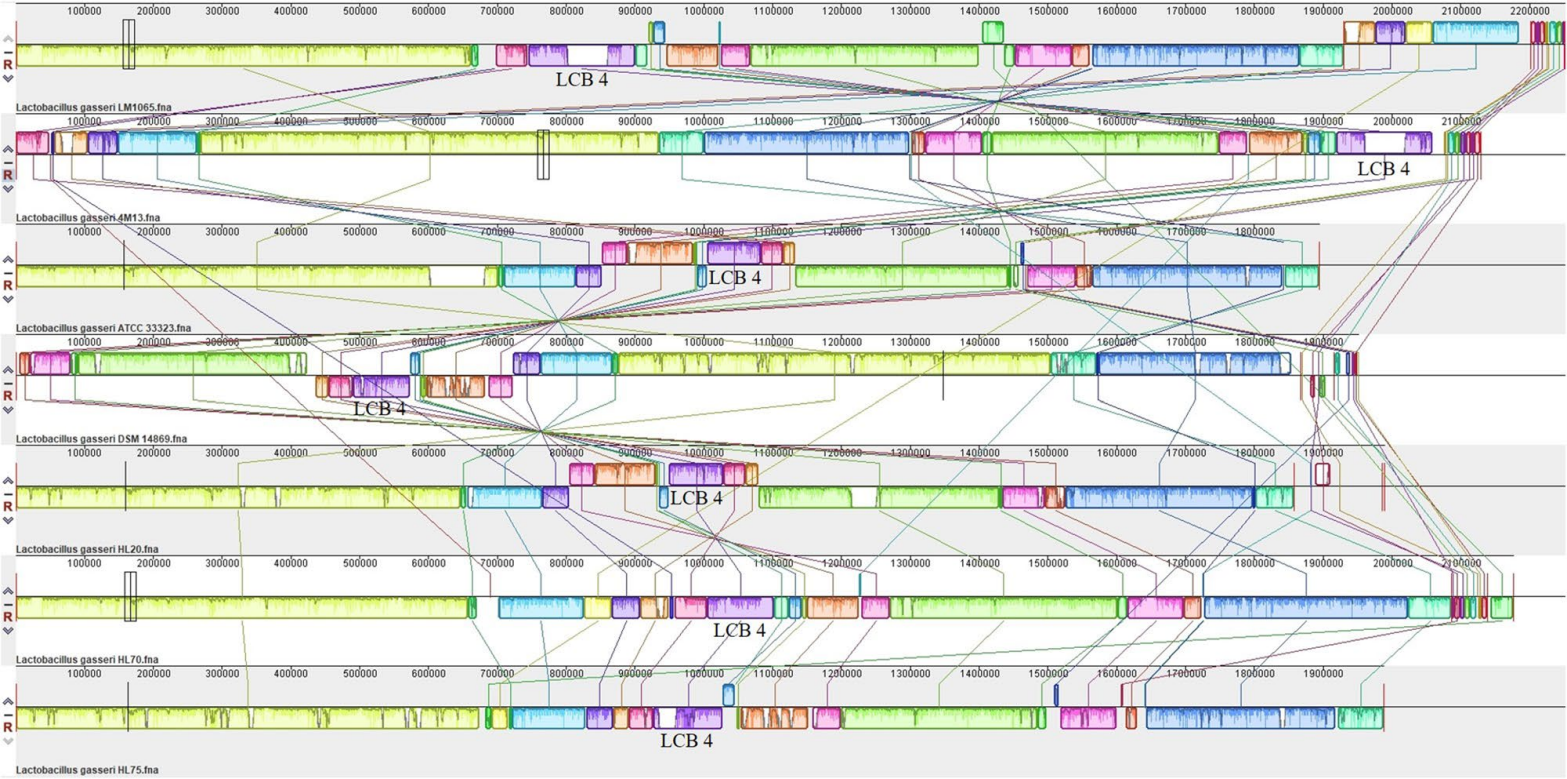
Multiple genome alignment and genomic comparison of *Lactobacillus gasseri*.

Figure 2 shows multiple alignment of the *L. gasseri* strains in relation to strains closet in homology using MAUVE. *L. gasseri* LM1065 was most similar to *L. gasseri* HL70 comparing to Locally Collinear Blocks (LCBs). A total of 27 LCBs were arranged in *L. gasseri* LM1065. Especially, LCB 4 showed difference in each *L. gasseri* such as length and alignment.

### Fatty acid composition of cell wall

Table S1 shows the fatty acid composition of the bacterial cell wall of *L. gasseri* LM1065. Thirteen fatty acids were investigated in *L. gasseri* LM1065. Oleic acid and *cis*-10-nonadecenoic acid were measured 68.95 and 16.31%, respectively, in *L. gasseri* LM1065. Most of the cellular fatty acids in *L. gasseri* LM1065 were unsaturated fatty acids (USFA, 87.96%), whereas saturated fatty acids (SFA) accounted for 12.04%. The USFA/SFA ratio was 7.30.

### Resistance to pepsin and bile salt

The viable cell numbers and survival rates of *L. gasseri* LM1065 under artificial gastric conditions are shown in Table 2. *L. gasseri* LM1065 showed an 88.97% survival rate in artificial pepsin for 2 h. The survival rate of *L. gasseri* LM1065 was not significantly different from that of *L. rhamnosus* ATCC 53103 (92.46%). Despite its high acid tolerance, *L. gasseri* LM1065 showed mild resistance in 0.05 and 0.1% of bile salt conditions. The changes of viable cells were showed 7.95 to 6.96 Log CFU/mL and 7.96 to 7.93 Log CFU/mL in 0.1% and 0.05% of bile salt, respectively.

**Table 2:** Acid tolerance, bile salt tolerance, auto-aggregation, hydrophobicity and adherence to intestinal epithelial cell ability of *Lactobacillus gasseri* LM1065.

Acid tolerance (0.3% pepsin)			
Strain	0 h	24 h	Survival rate (%)
ATCC 53103	8.30 ± 0.05	8.27 ± 0.01	92.46 ± 4.23
LM1065	7.66 ± 0.00	7.60 ± 0.04	88.97 ± 9.24
Bile salt tolerance (0.05% bile salt)			
Strain	0 h	24 h	Survival rate (%)
ATCC 53103	8.80 ± 0.09	8.98 ± 0.03	151.58 ± 12.33
LM1065	7.96 ± 0.11	7.93 ± 0.10	94.60 ± 22.80
Bile salt tolerance (0.1% bile salt)			
Strain	0 h	24 h	Survival rate (%)
ATCC 53103	9.14 ± 0.05	9.00 ± 0.05	71.36 ± 7.92
LM1065	7.95 ± 0.05	6.96 ± 0.02	10.26 ± 0.45
Bile salt tolerance (0.2% bile salt)			
Strain	0 h	24 h	Survival rate (%)
ATCC 53103	8.26 ± 0.04	8.03 ± 0.09	59.12 ± 12.04
LM1065	7.81 ± 0.14	4.78 ± 0.05	0.09 ± 0.01
Bile salt tolerance (0.3% bile salt)			
Strain	0 h	24 h	Survival rate (%)
ATCC 53103	8.64 ± 0.02	8.30 ± 0.09	46.49 ± 9.24
LM1065	7.81 ± 0.07	2.95 ± 0.17	< 0.01
Auto-aggregation (%)			
ATCC 53103		LM1065	
4 h	24 h	4 h	24 h
14.52 ± 4.59	48.46 ± 0.16	21.52 ± 5.09	61.21 ± 2.70**
Hydrophobicity (%)			
ATCC 53103		LM1065	
64.23 ± 5.27		61.55 ± 2.62	
Adherence ability (%)			
ATCC 53103		LM1065	
1.77 ± 0.33		2.02 ± 0.29	

Data are shown as means ± standard deviations of three independent experiments.

Significant difference is compared to *Lacticaseibacillus rhamnosus* ATCC 53103 (*P* < 0.01).

### Auto-aggregation, hydrophobicity, and adherence to HT-29 cells

Table 2 shows the auto-aggregation, hydrophobicity, and adherence to human epithelial cells of *L. gasseri* LM1065. *L. gasseri* LM1065 showed higher auto-aggregation ability (61.21%) than *L. rhamnosus* ATCC 53103 (48.46%) (*P* < 0.01). The hydrophobicity to hexadecane and the ability to adhere to HT-29 were measured 61.55% and 2.02%, respectively. These properties are not significantly different from those of *L. rhamnosus* ATCC 53103.

### Enzyme activity

API ZYM shows the intrinsic enzymatic properties of microorganisms using 19 different substrates. *L. gasseri* LM1065 showed 11 enzyme activities, including alkaline phosphatase, esterase, leucine arylamidase, cystine arylamidase, acid phosphatase, naphthol-AS-BI-phosphohydrolase, α-galactosidase, β-galactosidase, α-glucosidase, β-glucosidase, and N-acetyl-β-glucosaminidase (Table S2).

#### Safety evaluation

*L. gasseri* LM1065 showed antibiotic susceptibility according to ESFA guidelines (ampicillin, 0.5 μg/mL; chloramphenicol, 4 μg/mL; clindamycin, 0.5 μg/mL; erythromycin, 0.25 μg/mL; gentamicin, 4 μg/mL; kanamycin, 64 μg/mL; streptomycin, 16 μg/mL; tetracycline, 2 μg/mL; vancomycin, 0.5 μg/mL). The *L. gasseri* LM1065 was not detected antibiotics resistance and potential antibiotics resistance genes (ARGs). Putative virulence factors were not predicted by genome sequencing. Hemolysis was not detected on the blood agar (γ-hemolysis) (Table 3).

**Table 3:** Antibiotic resistance, CRISPR-Cas, plasmid seqeunces, virulence factors, and hemolysis in *Lactobacillus gasseri* LM1065.

Antibiotic sensitivity							
Antibiotics	Cut-off value (μg/mL)^a^	Minimum inhibitory concentration (μg/mL)	Potential antibiotic resistnace gene^b^				
Ampicillin	1	0.5	Not detected				
Chloramphenicol	4	4	Not detected				
Clindamycin	1	0.5	Not detected				
Erythromycin	1	0.25	Not detected				
Gentamicin	16	4	Not detected				
Kanamycin	64	64	Not detected				
Streptomycin	16	16	Not detected				
Tetracyline	4	2	Not detected				
Tylosin	Not required	-	Not detected				
Vancomycin	2	0.5	Not detected				

CRISPR^c^							
Location	Start	End	Orientation	Consensus repeat	Number of repeats	Number of spacers	Evidence level
Contig 1	1,070,760	1,071,191	Unknown	GATCTAAGCCTTATTGATCTAACAACCATCTAAAAC	6	6	4
plasmid	37,182	37,284	Unknown	CTTTCGATTTTCGAAATTCCGCTCGTAGCAAGGGTTT	2	1	1

Cas^c^							
Location	Type of Cas gene	Start	End				
Contig 1	csn2_TypeIIA	1,071,224	1,071,892				
Contig 1	cas2_TypeI-II-III	1,071,889	1,072,197				
Contig 1	cas1_TypeII	1,072,172	1,073,080				
Contig 1	cas9_TypeII	1,073,286	1,077,446				

Plasmid sequence type^d^		Virulence gene^e^		Hemolysis			
RepA_N	Not detected	Gelatinase (*gelE*)	Not detected	Type of hemolysis	Gamma		
Rep1	Not detected	Hyaluronidase (*hyl*)	Not detected				
Rep2	Not detected	Aggregation substance (*asa1*)	Not detected				
Rep3	Not detected	Entericoccal surface protein (*esp*)	Not detected				
RepL	Not detected	Cytolysin (*cylA*)	Not detected				
Rep_trans	Not detected	Cyototoxin K (*cytK*)	Not detected				
NT_Rep	Not detected	Enterotoxin (*nhe*)	Not detected				
Inc18	Not detected	Hemolysin (*hbl*)	Not detected				
		Serine protase (*sprE*)	Not detected				

^a^Cut-off values are *Lactobacillus acidophilus* group established by the European Food Safety Authority (EFSA).

^b^Antibiotic resistance genes were predicted using the comprehensive antibiotic resistance database (CARD) and ResFinder.

^c^Plasmid sequence types were predicted using the PlasmidFinder.

^d^CRISPR-Cas were predicted using the CRISPRCasFinder.

^e^Virulence genes were predicted by virulence factor database (VFDB).

#### Mobile genetic elements and genomic island

Mobile genetic elements (MGEs) in *L. gasseri* LM1065 were shown in Table S3. Total 7 of prophages, 2 of integrative and conjugative elements (ICEs), 57 of transposon insertion-sequence (IS) or IS cluster were detected in *L. gasseri* LM1065. In genomic island (GI) analysis, pathogenicity island and antibiotics resistance island were not found in *L. gasseri* LM1065. Plasmid sequences were not detected in *L. gasseri* LM1065. Clustered Regularly Interspaced Short Palindromic Repeats (CRISPR) and CRISPR-associated protein (Cas) which protect against MGEs were detected in *L. gasseri* LM1065.

#### Inhibition of *Candida albicans*

**Figure 3:**
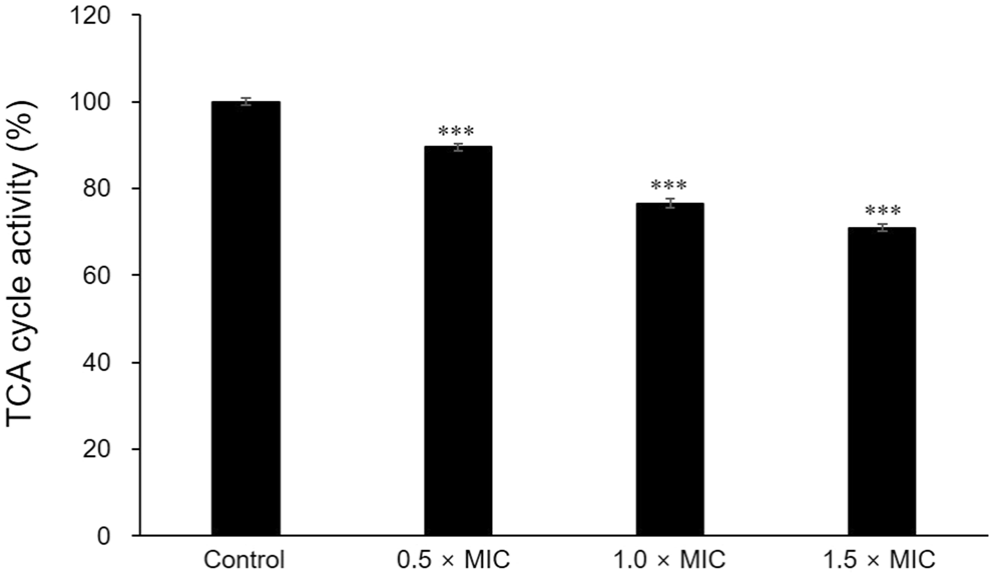
Tricarboxylic acid cycle activity in *Candida albicans* ATCC 11006 treated by cell free supernatant. Significant differences within test groups were analyzed Tukey’s range tests (*P* < 0.001).

**Figure 4:**
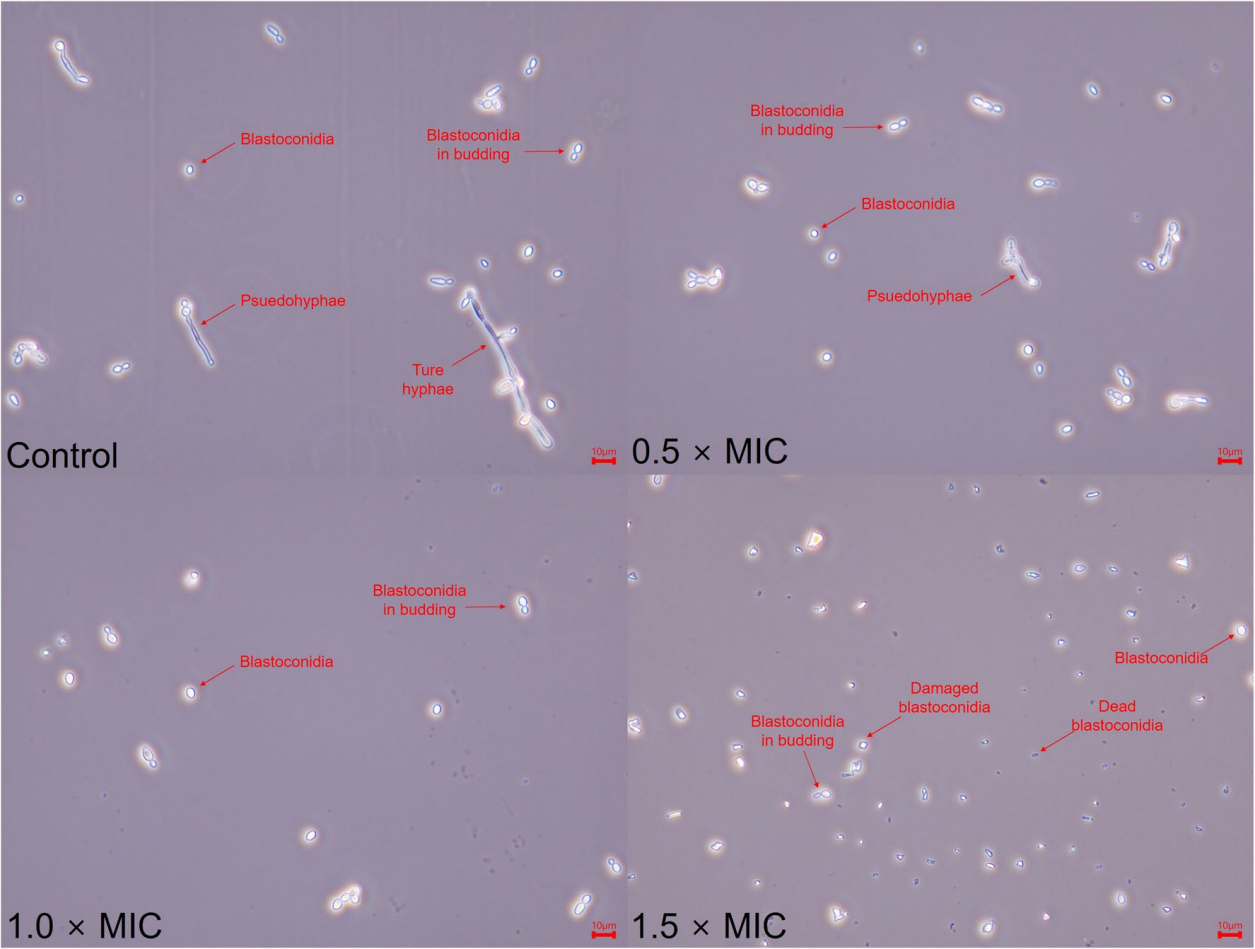
Microscopic features of *Candida albicans* ATCC 11006 treated by cell free supernatant (observed at the magnification of 400 ×).

Table 4 shows MIC and fungistatic effect of *L. gasseri* CFS. The MIC of CFS was evaluated 50%. To investigate fungistatic effect of CFS, *C. albicans* was incubated with different concentrations of CFS. Non-treated *C. albicans* grew up to 7.20 Log CFU/mL while CFS treated *C. albicans* was inhibited growth to 3.89 Log CFU/mL for 48 h. The decrease of growth rate was also detected by TCA cycle inhibition. CFS inhibited TCA cycle of *C. albicans* to 10.45, 23.46 and 29.02% in 0.5, 1.0 and 1.5 × MIC, respectively (*P* < 0.01) (Fig. 3). Moreover, CFS inhibited yeast to hyphae transition and damaged to blastoconidia in *C. albicans* (Fig. 4).

**Table 4:** Minimum inhibitory concentration (MIC) and growth inhibitory ability of *Lactobacillus gasseri* LM1065 cell free supernatant against *Candida albicans*.

Minimum inhibitory concentration (%)	Viable cell (Log CFU/mL)		
50	Concentration	Time (h)	
		0	48
	Control	3.94 ± 0.19^a^	7.20 ± 0.06^d^
	0.5 × MIC		6.79 ± 0.08^c^
	1.0 × MIC		5.83 ± 0.08^b^
	1.5 × MIC		3.89 ± 0.09^a^

Data are shown as means±standard deviations of three independent experiments.

Significant differences within test groups were analyzed by Duncan’s multiple range tests.

## Discussion

Probiotics are live microorganisms that contribute to host health when adequate amounts are consumed^2,15–17^. Probiotics are required to survive through the GIT and colonize the small intestine and colon for a long period^6^ with a safety guarantee^18,19^. *L. gasseri* LM1065 exhibited survival under gastric conditions, enzyme production, colonization and adhesion ability without antibiotic resistance, hemolysis, and putative virulence factors compared to functional gene annotation.

Probiotics are exposed to various types of stress during freeze-drying^20^, containing a food matrix and passing through the GIT^16^. These external stresses can damage cell membranes, induce the release of internal beneficial enzymes, and inhibit the colonization of the intestine^6,20^. In the present study, stress-responsive functional genes, including acid shock proteins, were investigated in *L. gasseri* LM1065 (Table 1). *L. gasseri* LM1065 showed high acid and mild bile salt tolerance (Table 2). Tang et al.^21^ reported that the bile salt concentrations vary from 0.03 to 0.3% in the small intestine during food digestion. *L. gasseri* LM1065 can survive in low concentrations of bile salts within the small intestine. Li et al.^17^ suggested that mild bile salt tolerance results from the microenvironment of the microbial niche. *Lactobacillus taiwanensis*, isolated from Peyer’s patches, also showed mild tolerance to bile salt because Peyer’s patches show mild bile salt conditions compared to other small intestinal sites. These properties were also observed in *Limosilactobacillus fermentum* 4LB16 and 10LB1 isolated from the human vaginal tract^22^.

In general, bacterial adhesion to the human intestinal tract is mediated by cell surface components. In *Lactobacillus*, surface-layer proteins, cell wall-anchored mucus-binding proteins, cell-surface collagen-binding proteins, and mannose-specific adhesins have been reported to adhere to host molecules and mechanisms^23^. The cell wall-anchored protein (CWAP) contains five amino acid motifs, LPXTG, which is constructed from Leu-Pro-any amino acid-Thr-Gly^24^. Gram-positive bacteria utilize sortase to cleave surface proteins, particularly the Thr-Gly residue of LPXTG. The cleaved threonine residue mediates cell wall attachment^25^. Zhang et al.^23^ studied the transformation of LPXTG into *Lactococcus lactis*, which confers adhesion to human epithelial cells. *L. gasseri* LM1065 showed higher auto-aggregation and adherence than *L. rhamnosus* ATCC 53103 (Table 2). LPXTG and sortase were predicted in *L. gasseri* LM1065 (Table 1), and these genetic properties contribute to its essential probiotic properties.

Gut-microbiomes produce enzymes that behave in complementary ways in human metabolism^26^. In the gut microbiome niche, LAB play critical roles in digestion, nutrient absorption, and improvement of nutritional value using numerous enzymes^27^. Most represent enzymes in *Lactobacillus* are lactase, β-galactosidase, glycosidase, protease, lipase, esterase and phytase^26,27^. Otherwise β-glucuronidase, which is released by *Escherichia*, *Clostridium*, and *Staphylococcus*^26^, is associated with potential carcinogenic metabolite conversion^19,26^. *L. gasseri* LM1065 released 11 enzymes, including β-galactosidase whereas β-glucuronidase activity was not found (Table S2). β-Galactosidase is an important biotechnological source in the food industry. β-Galactosidase is applied to ice cream to prevent undesirable crystallization and improve its creaminess. In addition, β-galactosidase is used in bakeries to improve sweetness^27^. Lactose intolerance occurred in a person lacking β-galactosidase in the intestine and caused a lack of β-galactosidase accumulation of lactose. Excessive lactose affects osmotic pressure, resulting in digestive disorders^26^. Thus, β-galactosidase produced by gut-microbes attenuate digestive disorders in lactose intolerance consumers^26,27^. Interestingly, *L. gasseri* LM1065 was predicted to contain β-galactosidase and glycoside hydrolase family 1 involved in lactose hydrolysis (Table 1).

In the host immune system, pattern recognition receptors (PRRs) play a critical role in recognizing pathogens from the external environment via pathogen-associated molecular patterns (PAMPs)^28,29^. The cell wall of *C. albicans* consists of two parts: mannosylated protein (outer layer) and β-1,3-glucan with underlying chitin (inner layer). Dectin-1 is a major PRR that recognizes β-1,3-glucan as a PAMP^29^. β-1,3-glucan is not only a PAMP but also a biofilm component that resists stress^30^. Therefore, β-1,3-glucan regulation is considered an effective strategy for the treatment of *C. albicans* and VVC^30,31^. *L. gasseri* LM1065 was predicted to contain the β-glucanase gene (Table 1), which inhibits the growth and hyphae transition of *C. albicans* ATCC 11006 (Fig. 4). Additionally, *L. gasseri* LM1065 was predicted to produce gassericin T (Table 1 and Fig. 1E).

## Conclusions

*L. gasseri* LM1065, isolated from human breast milk, has essential probiotic properties, including resistance to gastric conditions and adherence to intestinal cells. *L. gasseri* LM1065 satisfied safety requirements, including antibiotic resistance and putative virulence factor genes. Additionally, *L. gasseri* LM1065 was suggested as a probiotic for women, as the bacteria can inhibit candidiasis by suppressing the TCA cycle in *C. albicans* and blocking its transition to hyphae.

## Materials and methods

### Subjects and isolation of *Lactobacillus gasseri* LM1065

The collection of human breast milk were approved by Institutional Review Board of the Lactomason according to Enforcement Decree of Bioethics and Safety Act in Korea. All donors provided written informed consent before enrollment in the study and all methods were carried out in accordance with the Declaration of Helsinki. Three human breast milk samples were donated by three healthy women (25–40 years old) living in Gyeongsangnam-do on May 24, 2017. The participants did not have any underlying conditions and took any antibiotics or probiotics prior to the study for at least three months^18^. Human breast milk specimens were placed in a sterile container and diluted 10 times with phosphate-buffered saline (PBS, pH 7.2). The diluted specimens were spread on de Man–Rogosa–Sharpe (MRS) agar (for *Lactobacillus*) (BD Biosciences, Franklin Lakes, NJ, USA), M17 agar (for lactic *Streptococcus* and *Lactococcus*) (MBcell, Seoul, South Korea), and *Bifidobacterium* selective agar (for *Bifidobacterium* spp.) (MBcell). The spread agar plates were then incubated at 37 °C for 48 h. After 48 h, colonies isolated from MRS agar were spread on bromocresol purple (BCP) agar, and yellow colonies on BCP agar were further purified in newly prepared MRS agar until a single colony was obtained. Single and pure colonies were enriched in MRS broth for Gram staining and catalase reactions. The isolate was identified as a Gram-positive catalase-negative rod-type strain. The isolated strain was named LM1065 based on the institution’s naming system and identified by 16S rRNA sequencing as *L. gasseri*. *L. gasseri* LM1065 was stored in MRS containing 20% glycerol at − 80 °C until use^32^.

### Microorganisms and conditions

*L. rhamnosus* ATCC 53103 and *C. albicans* ATCC 11006 were purchased from the American Type Culture Collection (ATCC). *L. gasseri* LM1065 and *L. rhamnosus* ATCC 53103 were cultured in MRS broth at 37 °C with aerobic condition and sub-cultured three times every 12 h until use. *C. albicans* ATCC 11006 was cultured in yeast mold (YM) broth at 24 °C for 48 h with aerobic condition until use.

### Extraction of genomic DNA and genome analysis

*L. gasseri* LM1065 was harvested by centrifugation at 12,000 rpm at 4 °C for 10 min. The harvested bacterial cells were washed with PBS and genomic DNA (gDNA) was extracted. gDNA was extracted from *L. gasseri* LM1065 using the TaKaRa MiniBEST Bacteria Genomic DNA Extraction Kit (Takara Bio, Kusatsu, Japan) according to the manufacturer’s guidelines.

A total of 5 μg of the gDNA sample was used for library preparation. A DNA library was constructed and sequenced using single molecular real-time (SMRT) sequencing technology (Pacific Biosciences, Menlo Park, CA, USA). The SMRTbell library preparation was performed with the SMRTbell Template Prep Kit 1.0, and DNA/Polymerase Biding kit P6. The SMRT library was sequenced using 1 SMRT cell using C4 chemistry (DNA sequencing Reagent 4.0) and 240-min movies were captured for each SMRT cell using the PacBio RS II system by Insilicogen (Yongin, South Korea). The genome coverage (depth of coverage) was 195 × and HiFi long-read sequencing perform for analysis.

De novo assembly was performed in the hierarchical genome assembly process (HGAP) including consensus polishing workflow using Quiver, and 1,929,825 bp of N50 contig and 2,251,884 bp of total contig were obtained. Sequence alignment and contig formation were performed using MUMmer 3.5. The coding sequence was predicted using GLIMMER 3.0, and the GO analysis was performed using Blast2GO^33^. The plasmid was predicted using nucleotide BLAST and bacteriocin gene clusters were predicted and visualized by antiSMASH bacterial version 7.0.0^34^.

Multiple alignment and genomic comparison were analyzed using MAUVE^35^.

### Phylogenetic analysis based on orthologous gene and comparing of average nucleotide identity

Phylogenetic relationships of *L. gasseri* LM1065 were constructed based on ortholog gene sequences. The whole genome sequences of *Lactobacillus* and *Lactiplantibacillus* were obtained from the National Center for Biotechnology Information (NCBI) database. Ortholog analysis was performed using OrthoFinder v2.5.4 and species tree was inferred using STAG algorithm and rooted using STRIDE algorithm in OrthoFinder^36^. The species tree was illustrated by Dendroscope 3^37^.

The ANI values were estimated using the OrthoANI algorithm in EzBioCloud (https://www.ezbiocloud.net/tools/ani)^38^. The result of ANI distance was generated using the heatmap plot function of the TBtools^39^.

### Cellular fatty acid analysis of *Lactobacillus gasseri* LM1065

Cellular fatty acid extraction of *L. gasseri* LM1065 was performed using the Bligh and Dyer method with modifications^20^. In brief, 200 μL of chloroform/methanol solution (2:1, v/v) and 300 μL of 0.6 M hydrochloric acid solution (in methanol) were added to 20 mg of lyophilized *L. gasseri* LM1065. The mixture was shaken vigorously for 2 min and then incubated at 85 °C for 60 min. The reaction mixture was cooled at 25 °C for 20 min, and fatty acid methyl esters (FAME) were extracted using *n*-hexane for 60–120 min. The FAME extracted layer (*n*-hexane layer) was transferred into a clear vial and stored at − 20 °C until analysis.

Cellular fatty acid analysis was performed using Gas Chromatography/Mass selective detector (GC/MSD). The GC/MSD system was composed of an Agilent 8890 gas chromatography system coupled with a 5977 B mass selective detector (MSD) and a 7693A automated liquid sampler (Agilent, Santa Clara, CA, USA). An Agilent J&W DB-FastFAME capillary column packed with cyanopropyl (30 m × 0.25 mm, 0.25 μm) was employed. The injection port temperature was 250 °C under constant flow, and 1 μL of the sample was injected using the split mode of 20:1. Ultrapure helium was used as the carrier gas at a flow rate of 1 mL/min. The initial oven temperature was 60 °C for 1 min, raised from 60 to 165 °C at a rate of 60 °C/min, held for 1 min at 165 °C, raised from 165 to 230 °C at a rate of 5 °C/min, and maintained for 3 min. The temperatures of the ion source and transfer line were 230 °C and 250 °C, respectively. Mass spectra were obtained using electron ionization (EI) at 70 eV and recorded *m/z* 40–550 of mass range. Methyl undecanoate was used as an internal standard^40^.

### Tolerance to pepsin and bile salt

The resistance properties of *L. gassseri* LM1065 to artificial gastric conditions were investigated in a previous study with modifications^2,15–17,21,22^. *L. gasseri* LM1065 was inoculated into MRS containing 0.3% pepsin (pH 2.5) or oxgall (0.05, 0.1, 0.2, and 0.3%) and incubated at 37 °C. After 2 and 24 h of incubation, viable cells in pepsin- and oxgall-containing MRS broths were measured by spreading on MRS agar. *L. rhamnosus* ATCC 53103 was used as the control.

### Auto-aggregation and cell surface hydrophobicity

*L. gasseri* LM 1065 was harvested by centrifugation at 12,000 rpm and 4 °C for 10 min and washed twice with PBS. The washed cells were resuspended in PBS and adjusted to an OD_600_ of 0.5.

To evaluate the auto-aggregation of *L. gasseri* LM1065, the adjusted bacterial suspension was allowed to stand and incubated at 37 °C. The upper suspension was collected (4 and 24 h), and the absorbance was measured at 600 nm. Auto-aggregation was calculated using the following equation:

$${\text{Auto}} - {\text{aggregation}} \left( \% \right) = \frac{{{\text{A}}_{0} - {\text{A}}_{{{\text{time}}}} }}{{{\text{A}}_{0} }} \times 100$$

where A_0_ is the initial absorbance (0.5), and A_time_ is the absorbance of the supernatant at 4 and 24 h, respectively^2,11^. *L. rhamnosus* ATCC 53103 was used as the control.

To evaluate the hydrophobicity of *L. gasseri* LM1065, 2 mL of the adjusted bacterial suspension was mixed with 1 mL hexadecane. The mixture was then allowed to stand at 25 °C for 30 min. After incubation, the aqueous phase was separated, and its absorbance was measured at 600 nm. The hydrophobicity was calculated using the following equation.

$${\text{Hydrophobicity}} \left( \% \right) = \frac{{{\text{A}}_{0} - {\text{A}}_{30} }}{{{\text{A}}_{0} }} \times 100$$

where A_0_ is the initial absorbance (0.5) and A_30_ is the absorbance of the aqueous phase at 30 min^18,40^. *L. rhamnosus* ATCC 53103 was used as the control.

### Adhesion to human intestinal epithelial cell

Adhesion ability of *L. gasseri* LM1065 was investigated in human intestinal epithelial cell (HT-29)^15,18,41^. HT-29 human intestinal epithelial cells were purchased from Korean Cell Line Bank (Seoul, South Korea). The cells were maintained in Roswell Park Memorial Institute (RPMI) 1640 medium (Gibco, Waltham, MA, USA) supplemented with 10% fetal bovine serum (FBS) and 1% penicillin–streptomycin solution at 37 °C in a humidified atmosphere containing 5% CO_2_. During incubation, the media were changed every 2–3 days, and cells were grown to 80% confluence. When HT-29 cells reached 80% confluence, the adherent cells were trypsinized with trypsin–EDTA solution (0.25%) and harvested by centrifugation. Harvested cells were seeded in 24-well plates (1 × 10^5^ cells/well) and incubated with changing media to form a monolayer. Activated *L. gasseri* LM1065 was diluted to approximately 8 Log CFU/mL, and the HT-29 monolayer was treated with diluted *L. gasseri* LM1065 for 2 h without antibiotics. After 2 h, non-adherent bacterial cells were washed using PBS, and adherent bacterial cells were collected using 1% (v/v) Triton-X solution. Adherent bacterial cells were spread on MRS agar and viable cells were estimated. *L. rhamnosus* ATCC 53103 was used as the control.

### Enzymatic profile of *Lactobacillus gasseri* LM1065

The intrinsic enzyme activities of *L. gasseri* LM1065 were estimated using API ZYM, according to the manufacturer’s guidelines (bioMérieux, Marcy-l’Étoile, France).

#### Safety assessments

Safety assessments were measured based on antibiotic resistance^18,22,42^, analysis of ARGs, virulence genes^19,42^, and hemolysis^18,22,41^.

Antibiotic resistance of *L. gasseri* LM1065 was evaluated according to the European Food Safety Authority (EFSA) guidelines. Ampicillin, erythromycin, gentamicin, tetracycline, streptomycin, vancomycin, chloramphenicol, kanamycin, and clindamycin susceptibilities were measured by cut-off values using ETEST® strips (bioMérieux).

ARGs and virulence genes were predicted using a nucleotide database. The draft genome sequence of *L. gasseri* LM1065 was performed to identify genetic variations. The Comprehensive Antibiotic Resistance Database (CARD) (https://card.mcmaster.ca/)^7,19^ and ResFinder (https://cge.food.dtu.dk/services/ResFinder/)^42^ were used for genome-based analysis of ARGs. Virulence genes were analyzed by comparison with the Virulence Factor Database (VFDB) (http://www.mgc.ac.cn/VFs/main.htm)^42^.

Hemolysis activity was determined using Columbia blood agar (Oxoid, Basingstoke, United Kingdom) containing 5% sheep blood.

#### Mobile genetic elements and genomic island

The MGEs and genomic island were measured for predicting horizontal gene transfer. CRISPR-Cas were investigated by CRISPRCasFinder (https://crisprcas.i2bc.paris-saclay.fr/CrisprCasFinder/Index)^43^. Plasmid sequences were detected using PlasmidFinder (https://cge.food.dtu.dk/services/PlasmidFinder/)^43^. Prophage, IS, and GI were analyzed using VRprofile2 (https://tool2-mml.sjtu.edu.cn/VRprofile/home.php)^44^.

#### Minimum inhibitory concentration and fungistatic effect of *Lactobacillus gasseri* LM1065

The cell-free supernatant (CFS) of *L. gasseri* LM1065 was prepared using a 0.45 μm cellulose acetate membrane filter. To determine the minimum inhibitory concentration (MIC), the CFS was diluted in a 96-well plate using YM broth (MBcell, Seoul, South Korea). After dilution, approximately 6 Log CFU/mL of *C. albicans* ATCC 11006 was added to each well and further incubated at 24 °C for 48 h. MIC was determined as the lowest concentration that did not show *C. albicans* growth visually^45^. To measure the fungistatic effect of CFS, approximately 7 Log CFU/mL of *C. albicans* ATCC 11006 was treated with different CFS concentrations (0.5, 1.0, and 1.5 × MIC) and incubated at 24 °C for 48 h. After incubation, *C. albicans* was spread on YM agar, further incubated at 24 °C, and viable cells were counted.

#### Tricarboxylic acid cycle inhibition and microscopic observation

Tricarboxylic acid (TCA) cycle activity in CFS-treated *C. albicans* ATCC 11006 was measured using an iodonitrotetrazolium chloride (INT; Sigma-Aldrich, MO, USA) solution^44^. Briefly, approximately 7 Log CFU/mL of *C. albicans* ATCC 11006 was treated with 0.5, 1.0 and 1.5 × MIC of CFS and incubated for 24 h. After incubation, *C. albicans* was collected by centrifugation at 3,000 rpm at 4 °C for 10 min and washed twice with PBS. The harvested *C. albicans* was diluted to an OD_600_ of 0.1, and INT solution (1 mM final concentration) was added. The INT solution-treated *C. albicans* cells were incubated at 37 °C for 30 min. The TCA cycle was assessed by measuring the absorbance of formazan at 630 nm.

For microscopic observation, the harvested cells were stained with 0.4% trypan blue solution (Gibco). The morphology of the CFS-treated *C. albicans* was observed using a BX53 biological microscope (Olympus, Tokyo, Japan). All observations were performed at a total magnification of 400 × . Microscopic images were obtained using the eXcope software. Morphological information was obtained from a previous study^46^.

#### Statistical analysis

Statistical analyses were performed using the SPSS Statistics version 18 software (IBM, Armonk, NY, USA). Mean values were analyzed using the *t*-test and one-way analysis of variance (ANOVA) followed by Duncan’s multiple range tests and Tukey's range test at *P* < 0.05.

#### Ethical consideration

The collection of human breast milk were approved by Institutional Review Board of the Lactomason according to Enforcement Decree of Bioethics and Safety Act in Korea. All donors signed an informed consent form before enrollment in the study and voluntarily provided samples for only research purpose in accordance with the Declaration of Helsinki.

## Supplementary Information

Supplementary Information.

## Data Availability

The draft genome sequence has been deposited at DDBJ/ENA/GenBank under the accession JAQOUF000000000 (https://www.ncbi.nlm.nih.gov/nuccore/JAQOUF000000000). The authors confirm that the data of this study are available within the article.
