# Supplementary material for: Draft genome sequence and probiotic functional property analysis of Lactobacillus gasseri LM1065 for food industry applications
Source: Sci Rep. 2023 Jul 27;13:12212. doi: 10.1038/s41598-023-39454-2 (PMC10374649; doi:10.1038/s41598-023-39454-2)
Supplement: Supplementary file 1 — Supplementary Information. [file 41598_2023_39454_MOESM1_ESM.docx]

**Appendix A (Supplementary information)**

**Draft genome sequence and probiotic functional property analysis of *Lactobacillus gasseri* LM1065 for food industry applications**

**
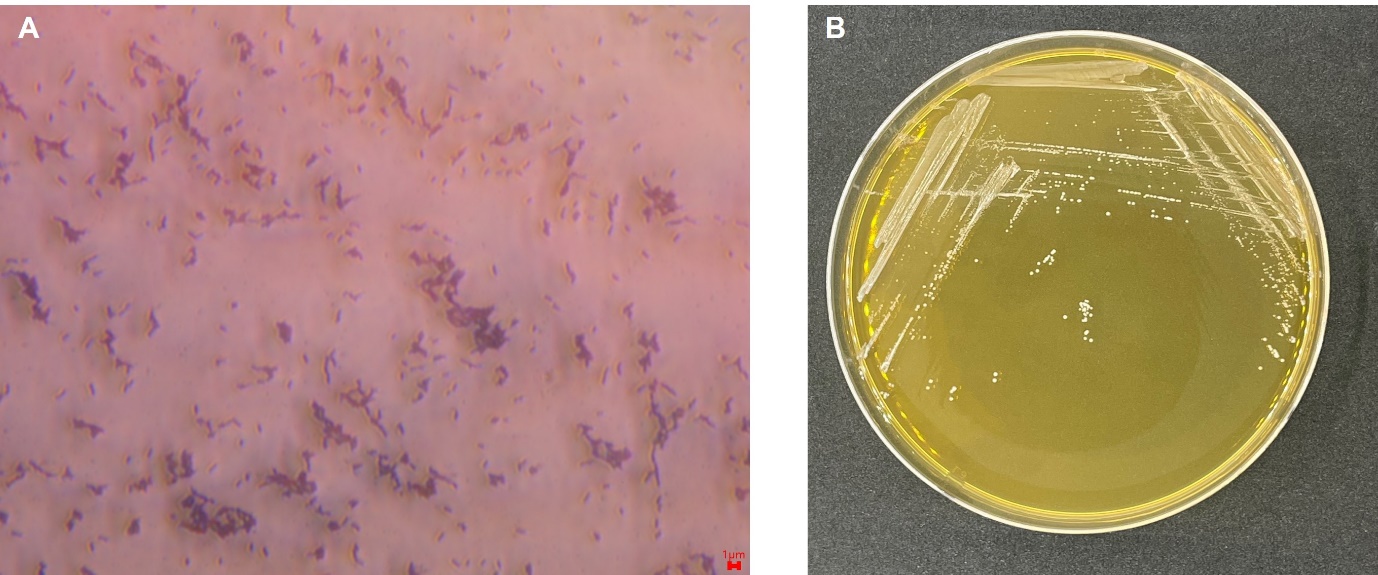
**

**Fig. S1.** Microscopic observation and colony morphology of *Lactobacillus gasseri* LM1065. **(A)** Gram stain observation of L. gasseri LM1065. Microscopic image was observed at the magnification of 1,000×. **(B)** Colony morphology of *L. gasseri* LM1065 on the MRS agar.

**Table S1.** Cellular fatty acid composition of *Lactobacillus gasseri* LM1065

| Fatty acids | Contents (%)^a^ |
| --- | --- |
| Octanoate (C8:0) | 0.03±0.00 |
| Decanoate (C10:0) | 0.15±0.00 |
| Dodecanoate (C12:0) | 0.47±0.00 |
| Myristate (C14:0) | 0.39±0.00 |
| Pentadecanoate (C15:0) | 0.25±0.01 |
| Palmitate (C16:0) | 8.81±0.06 |
| Palmitoleate (C16:1n7c) | 0.82±0.01 |
| Heptadecanoate | 0.38±0.01 |
| Stearate | 1.57±0.01 |
| Oleate | 68.95±0.11 |
| Mehtyl *cis*-11-vaccenic acid ester | 1.21±0.03 |
| Linoleate | 0.67±0.02 |
| *cis*-10-Nonadecenoate | 16.31±0.07 |
| Saturated fatty acid | 12.04±0.39 |
| Unsaturated fatty acid | 87.96±2.69 |
| Unstaturated fatty acids/saturated fatty acids | 7.30 |

^a^Fatty acid contents were analyzed in 1 g of lyophilized *L. gasseri* LM1065.

**Table S2.** Enzyme activity of *Lactobacillus gasseri* LM1065

| Enzyme | Enzyme activity |
| --- | --- |
| Alkaline phosphatase | positive |
| Esterase (C4) | positive |
| Esterase lipase (C8) | negative |
| Lipase (C14) | negative |
| Leucine arylamidase | positive |
| Valine arylamidase | negative |
| Cystine arylamidase | positive |
| Trypsin | negative |
| α-Chymotrypsin | negative |
| Acid phosphatase | positive |
| Naphthol-AS-BI-phosphohydrolase | positive |
| α-Galactosidase | positive |
| β-Galactosidase | positive |
| β-Glucuronidase | negative |
| α-Glucosidase | positive |
| β-Glucosidase | positive |
| N-Acetyl-β-glucosaminidase | positive |
| α-Mannosidase | negative |
| α-Fucosidase | negative |

**Table S3.** Mobile genetic elements in *Lactobacillus gasseri* LM1065.

| Location | Mobile genetic elements | Details | Start | End | GC contents (%) | ARGs | Virulence gene |
| --- | --- | --- | --- | --- | --- | --- | --- |
| Contig 1 | Prophage | - | 664,536 | 710,324 | 35.21 | Not detected | Not detected |
| Contig 1 | Prophage | - | 781,694 | 859,644 | 35.13 | Not detected | Not detected |
| Contig 1 | Prophage | - | 876,581 | 949,000 | 35.47 | Not detected | Not detected |
| Contig 1 | Prophage | - | 1,084,046 | 1,108,960 | 29.35 | Not detected | Not detected |
| Contig 1 | Prophage | - | 1,401,207 | 1,469,129 | 35.66 | Not detected | Not detected |
| Contig 1 | Prophage | - | 1,685,128 | 1,701,894 | 33.20 | Not detected | Not detected |
| Contig 1 | ICEs | - | 26,868 | 39,888 | 32.22 | Not detected | Not detected |
| Contig 1 | ICEs | - | 1,305,685 | 1,336,639 | 34.28 | Not detected | Not detected |
| Contig 1 | Transposon-IS | ISLga1 | 8,853 | 9,902 | 37.56 | Not detected | Not detected |
| Contig 1 | Transposon-IS cluster | ISLge5 / ISLh7 / ISLhe1 | 30,164 | 36,757 | 32.19 | Not detected | Not detected |
| Contig 1 | Transposon-IS | ISLga1 | 119,651 | 120,700 | 37.56 | Not detected | Not detected |
| Contig 1 | Transposon-IS | ISLga1 | 164,222 | 165,271 | 37.46 | Not detected | Not detected |
| Contig 1 | Transposon-IS | ISLga1 | 185,875 | 186,924 | 37.46 | Not detected | Not detected |
| Contig 1 | Transposon-IS | ISLga1 | 217,657 | 218,706 | 37.56 | Not detected | Not detected |
| Contig 1 | Transposon-IS | ISLga1 | 246,315 | 247,364 | 37.56 | Not detected | Not detected |
| Contig 1 | Transposon-IS cluster | ISLga1 / ISLga1 | 281,691 | 285,970 | 34.35 | Not detected | Not detected |
| Contig 1 | Transposon-IS | ISLga1 | 296,052 | 297,101 | 37.56 | Not detected | Not detected |
| Contig 1 | Transposon-IS | ISLga1 | 383,837 | 384,886 | 37.46 | Not detected | Not detected |
| Contig 1 | Transposon-IS cluster | ISLga1 / ISLga1 / ISLga1 | 412,476 | 424,806 | 35.06 | Not detected | Not detected |
| Contig 1 | Transposon-IS | ISLga1 | 442,405 | 443,454 | 37.46 | Not detected | Not detected |
| Contig 1 | Transposon-IS | ISLga1 | 472,060 | 473,109 | 37.65 | Not detected | Not detected |
| Contig 1 | Transposon-IS | ISLga1 | 507,970 | 509,019 | 37.46 | Not detected | Not detected |
| Contig 1 | Transposon-IS | ISLga1 | 534,838 | 535,887 | 37.46 | Not detected | Not detected |
| Contig 1 | Transposon-IS | ISLga1 | 548,345 | 549,394 | 37.65 | Not detected | Not detected |
| Contig 1 | Transposon-IS | ISLga1 | 564,862 | 565,911 | 37.65 | Not detected | Not detected |
| Contig 1 | Transposon-IS | ISLga1 | 603,694 | 604,743 | 37.56 | Not detected | Not detected |
| Contig 1 | Transposon-IS | ISLga1 | 619,400 | 620,449 | 37.56 | Not detected | Not detected |
| Contig 1 | Transposon-IS | ISLga1 | 644,050 | 645,099 | 37.46 | Not detected | Not detected |
| Contig 1 | Transposon-IS cluster | ISLga1 / ISLga1 | 652,065 | 657,800 | 35.99 | Not detected | Not detected |
| Contig 1 | Transposon-IS cluster | ISLga1 / ISLga1 | 737,903 | 744,855 | 34.13 | Not detected | Not detected |
| Contig 1 | Transposon-IS | ISLga1 | 762,425 | 763,474 | 37.27 | Not detected | Not detected |
| Contig 1 | Transposon-IS cluster | ISLga1 / ISLga1 | 772,798 | 775,922 | 35.05 | Not detected | Not detected |
| Contig 1 | Transposon-IS | ISLga1 | 878,890 | 879,939 | 37.56 | Not detected | Not detected |
| Contig 1 | Transposon-IS | ISLga1 | 918,284 | 919,333 | 37.46 | Not detected | Not detected |
| Contig 1 | Transposon-IS | ISLga1 | 926,744 | 927,793 | 37.46 | Not detected | Not detected |
| Contig 1 | Transposon-IS cluster | ISLga1 / ISLga1 | 940,812 | 945,272 | 35.16 | Not detected | Not detected |
| Contig 1 | Transposon-IS | ISLga1 | 964,084 | 965,133 | 37.27 | Not detected | Not detected |
| Contig 1 | Transposon-IS | ISLga1 | 987,229 | 988,278 | 37.65 | Not detected | Not detected |
| Contig 1 | Transposon-IS | ISLga1 | 1,060,923 | 1,061,972 | 37.46 | Not detected | Not detected |
| Contig 1 | Transposon-IS | ISLga1 | 1,068,569 | 1,069,618 | 37.65 | Not detected | Not detected |
| Contig 1 | Transposon-IS cluster | ISLjo2 / ISLga1 / ISLga1 / ISLga1 / ISLga1 | 1,089,003 | 1,100,823 | 31.10 | Not detected | Not detected |
| Contig 1 | Transposon-IS | ISLga1 | 1,109,764 | 1,110,813 | 37.46 | Not detected | Not detected |
| Contig 1 | Transposon-IS | ISLga1 | 1,191,893 | 1,192,942 | 37.46 | Not detected | Not detected |
| Contig 1 | Transposon-IS | ISLga1 | 1,219,413 | 1,220,462 | 37.46 | Not detected | Not detected |
| Contig 1 | Transposon-IS | ISLga1 | 1,234,516 | 1,235,565 | 37.65 | Not detected | Not detected |
| Contig 1 | Transposon-IS | ISLga1 | 1,335,590 | 1,336,639 | 37.65 | Not detected | Not detected |
| Contig 1 | Transposon-IS | ISLga1 | 1,360,663 | 1,361,712 | 37.56 | Not detected | Not detected |
| Contig 1 | Transposon-IS | ISLga1 | 1,440,990 | 1,442,039 | 37.65 | Not detected | Not detected |
| Contig 1 | Transposon-IS | ISLga1 | 1,455,320 | 1,456,369 | 37.46 | Not detected | Not detected |
| Contig 1 | Transposon-IS | ISLga1 | 1,493,592 | 1,494,641 | 37.37 | Not detected | Not detected |
| Contig 1 | Transposon-IS cluster | ISLjo2 / ISLjo2 | 1,550,490 | 1,551,766 | 35.03 | Not detected | Not detected |
| Contig 1 | Transposon-IS | ISLga1 | 1,635,439 | 1,636,488 | 37.65 | Not detected | Not detected |
| Contig 1 | Transposon-IS | ISLga1 | 1,690,437 | 1,691,486 | 37.46 | Not detected | Not detected |
| Contig 1 | Transposon-IS | ISLga1 | 1,730,680 | 1,731,729 | 37.46 | Not detected | Not detected |
| Contig 1 | Transposon-IS | ISLga1 | 1,758,300 | 1,759,349 | 37.46 | Not detected | Not detected |
| Contig 1 | Transposon-IS | ISLga1 | 1,767,632 | 1,768,681 | 37.56 | Not detected | Not detected |
| Contig 1 | Transposon-IS cluster | ISLga1 / ISLga1 | 1,780,923 | 1,783,919 | 33.98 | Not detected | Not detected |
| Contig 1 | Transposon-IS | ISLga1 | 1,815,710 | 1,816,759 | 37.46 | Not detected | Not detected |
| Contig 1 | Transposon-IS | ISLga1 | 1,893,792 | 1,894,841 | 37.56 | Not detected | Not detected |
| Contig 1 | Transposon-IS cluster | ISLga1 | 1,913,867 | 1,916,774 | 34.16 | Not detected | Not detected |
| Contig 2 | Prophage | - | 65664 | 155,174 | 35.21 | Not detected | Not detected |
| Contig 2 | Transposon-IS cluster | ISLga1 / ISLga1 | 68,993 | 72,468 | 35.17 | Not detected | Not detected |
| Contig 2 | Transposon-IS | ISLga1 | 83,317 | 84,366 | 37.56 | Not detected | Not detected |
| Contig 2 | Transposon-IS | ISLga1 | 245,782 | 246,831 | 37.56 | Not detected | Not detected |
| Plasmid | Transposon-IS cluster | ISLhe61 / ISLhe61 | 1,382 | 2,822 | 36.88 | Not detected | Not detected |
| Plasmid | Transposon-IS | IS1165 | 39,636 | 40,220 | 47.60 | Not detected | Not detected |
